# Breast cancer and incidence of type 2 diabetes mellitus: a systematic review and meta-analysis

**DOI:** 10.1007/s10549-023-07043-6

**Published:** 2023-09-01

**Authors:** Nanna Jordt, Kasper Aalbæk Kjærgaard, Reimar W. Thomsen, Signe Borgquist, Deirdre Cronin-Fenton

**Affiliations:** 1 Department of Clinical Epidemiology, Department of Clinical Medicine, Aarhus University Hospital & Aarhus University, Aarhus, Denmark; 2 Department of Oncology, Aarhus University Hospital, Aarhus, Denmark

**Keywords:** Breast cancer, Type 2 diabetes mellitus, Meta-analysis, Endocrine therapy, Tamoxifen, Aromatase inhibitors

## Abstract

**Purpose:**

Breast cancer and its treatments may increase the risk of type 2 diabetes (T2D). We conducted a systematic review and meta-analysis to investigate the association between breast cancer and the incidence of T2D overall, and according to breast cancer treatments.

**Methods:**

We searched PubMed, Embase and references of relevant papers for studies on breast cancer, breast cancer treatment, and subsequent T2D risk. Using random-effects models, we calculated effect estimates and associated 95% confidence intervals of the association between breast cancer, adjuvant breast cancer treatments (i.e., endocrine therapy (tamoxifen, aromatase inhibitors, and combined) and chemotherapy), and subsequent T2D. We used funnel plots to assess publication bias.

**Results:**

Among 15 eligible studies, 10 reported on T2D risk after breast cancer, chemotherapy, or endocrine therapy; five studies investigated more than one association. Compared with patients without breast cancer, those with breast cancer and those who received any endocrine therapy had elevated risk of incident T2D (EE = 1.23, 95% CI = 1.13–1.33 and EE = 1.23, 95% CI = 1.16–1.32, respectively). Among breast cancer patients only, the risk of T2D was higher for those who received tamoxifen compared with those who did not receive tamoxifen (EE = 1.28, 95% CI = 1.18–1.38). Due to few studies, analyses investigating T2D risk after treatment with aromatase inhibitors or chemotherapy were inconclusive.

**Conclusion:**

Our findings suggest an elevated risk of T2D in breast cancer survivors, particularly after tamoxifen therapy. Further research is needed to determine the impact of aromatase inhibitors, and chemotherapy on the incidence of T2D after breast cancer.

**Supplementary Information:**

The online version contains supplementary material available at 10.1007/s10549-023-07043-6.

## Introduction

Breast cancer is the most frequently diagnosed malignancy in women. In 2023, ~ 2.3 million incident breast cancers will be diagnosed worldwide. Due to improvements in breast cancer diagnostics and treatment, the population of breast cancer survivors has increased. In 2020, there were about 7.8 million 5-year survivors of breast cancer globally [1]. This number is expected to continue to increase [2], highlighting the need for better understanding of the late effects of the disease and its treatments. One such late effect may be type 2 diabetes (T2D).

Observational studies point towards increased risk of T2D in breast cancer survivors [3–7]. Breast cancer and T2D share risk factors including obesity, inflammation, and altered endogenous hormones [8]. Still, T2D may also occur as a complication of breast cancer therapy [3, 4, 9, 10]. For example, glucocorticoid therapy given concomitantly with chemotherapy may increase T2D risk due to weight gain, reduced insulin synthesis, reduced insulin sensitivity, and hyperglycemia [3, 10]. Endocrine therapy modifies insulin sensitivity and may invoke a persistent excess risk of T2D [9]. Low estrogen levels in postmenopausal women are associated with increased risk of T2D [11, 12]. Yet, aromatase inhibitors—the endocrine therapy recommended for postmenopausal women—inhibit estrogen synthesis [13]. Evidence also suggests that tamoxifen impairs glucose homeostasis by promoting apoptosis of pancreatic β-cells [14]. Tamoxifen is also associated with reduced insulin sensitivity in premenopausal women with overweight [15]. If breast cancer is associated with elevated risk of developing T2D, it may be beneficial to screen breast cancer survivors for T2D to expedite diagnosis and improve prognosis. It may also prompt the initiation of preventive measures in subgroups of breast cancer survivors deemed at high risk of developing T2D.

A meta-analysis by Ye et al. aimed to investigate the incidence of T2D after breast cancer and endocrine therapy, suggesting elevated risk of T2D in breast cancer survivors [16]. Still, their study did not include all eligible studies. As such, the evidence to date on the impact of breast cancer and subsequent risk of T2D requires clarification.

We therefore conducted a systematic review and meta-analysis to collate evidence on the association between breast cancer and subsequent risk of T2D. We also evaluated this association according to the receipt of breast cancer treatments.

## Methods

### Search strategy and eligibility criteria

We conducted this study in accordance with The Preferred Reporting Items for Systematic Reviews and Meta-Analyses (PRISMA) [17] and the Conducting Systematic Reviews and Meta-Analyses of Observational Studies of Etiology (COSMOS-E) guidelines [18].

We performed a search for all eligible studies in MEDLINE (PubMed) and EMBASE from inception through May 2022. See Online Resource 1 for search terms. We also searched the reference lists of eligible articles.

We included all observational studies where (i) breast cancer or breast cancer treatment [chemotherapy or endocrine treatment (tamoxifen or aromatase inhibitors)] among breast cancer patients was included as an exposure and (ii) the outcome was incident T2D. We restricted to published papers written in English, Danish, Norwegian or Swedish.

### Study selection and data extraction

Two investigators (medical student N.J. and PhD-student K.K.) independently screened all articles by title and abstract to remove irrelevant studies. The results from all searches were inserted into Covidence (Teamsquare, Melbourne, Australia) and duplicates were removed. The same two investigators performed independent unblinded eligibility assessment of the retrieved publications and evaluated their eligibility for inclusion based on the afore-mentioned eligibility criteria.

The two investigators used a predefined data extraction form to extract the following data from the eligible studies: author, title, journal, publication year, study design, study period, study size, length of follow-up, inclusion criteria, exclusion criteria, data source, methods, age and sex distribution, exposure (treatment), comparator group, outcome(s), risk estimate used, main results, and potential confounders adjusted for. When effect estimates (EEs) were not available, the investigators extracted the raw data. We also retrieved 95% confidence intervals (CI) as measures of precision.

### Diabetes mellitus outcome definition

Diabetes mellitus in the studies was ascertained via diagnostic codes, prescriptions for antidiabetic medication, and/or blood glucose testing. For studies that did not specify which type of diabetes mellitus was examined, we assumed it was T2D if the women were diagnosed with diabetes mellitus as adults (at age 18 years or older).

### Study quality and risk of bias assessment

Since there is no consensus regarding how to assess study quality and risk of bias, we followed the COSMOS-E guidelines [18]. Consequently, we evaluated the studies qualitatively by considering how thoroughly the authors reported methods and results as well as considering the potential for selection and information bias, and confounding within the studies.

### Statistical analyses

EEs for each outcome were pooled using a random-effects model with the Restricted Maximum Likelihood (REML) method and illustrated using forest plots. Statistical heterogeneity was assessed using I^2^ statistics.

Measures of relative risk [risk ratios (RR), odds ratios (OR), and hazard ratios (HR)] were considered equivalent. As the outcome is rare, and the studies investigated the same exposure and outcome, we considered it reasonable to compare the ratios [19]. In studies where no relative risk estimate was provided, RR and 95% CIs were calculated from raw data.

The estimates for breast cancer and risk of developing T2D were pooled in one analysis, while the effects of endocrine therapy overall, tamoxifen and aromatase inhibitors were examined in separate analyses. For each of these treatments, breast cancer patients were compared to breast cancer patients who did not use the treatment and/or non-cancer referents, depending on the data available from the articles.

Last, we used funnel plots to visually assess publication bias across studies.

Statistical analyses were performed using R Statistical Software [version 4.1.3 (2022-03-10); The R Foundation for Statistical Computing].

## Results

### Systematic literature search

**Fig. 1:**
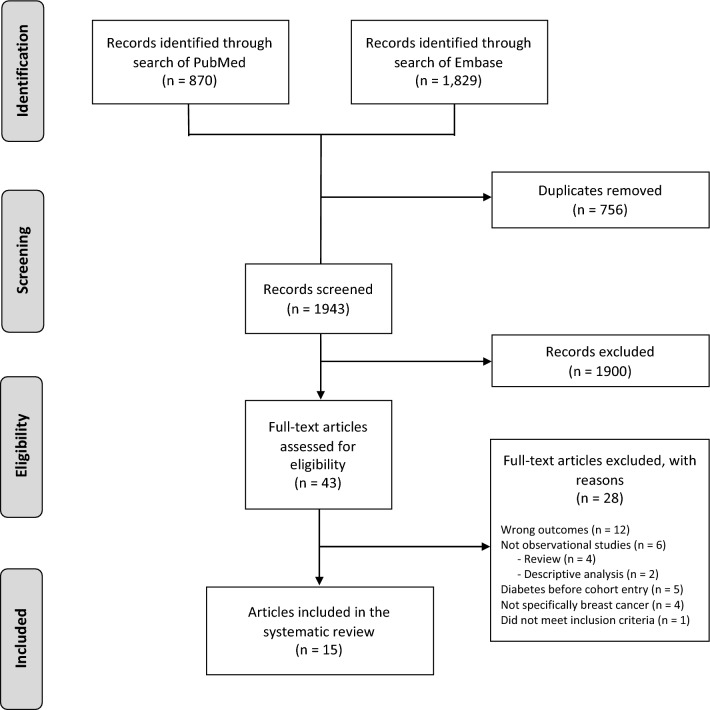
Flowchart of literature search

Our systematic search in PubMed and EMBASE yielded 2699 studies, of which 756 were duplicates and 1900 were excluded based on title or abstract alone. The full text of the remaining 43 studies was examined. Of these, 28 studies did not meet the eligibility criteria and were thus discarded (Fig. 1). When searching reference lists, we found no additional studies. Our systematic review therefore included a total of 15 studies [3–7, 9, 20–28].

**Fig. 2:**
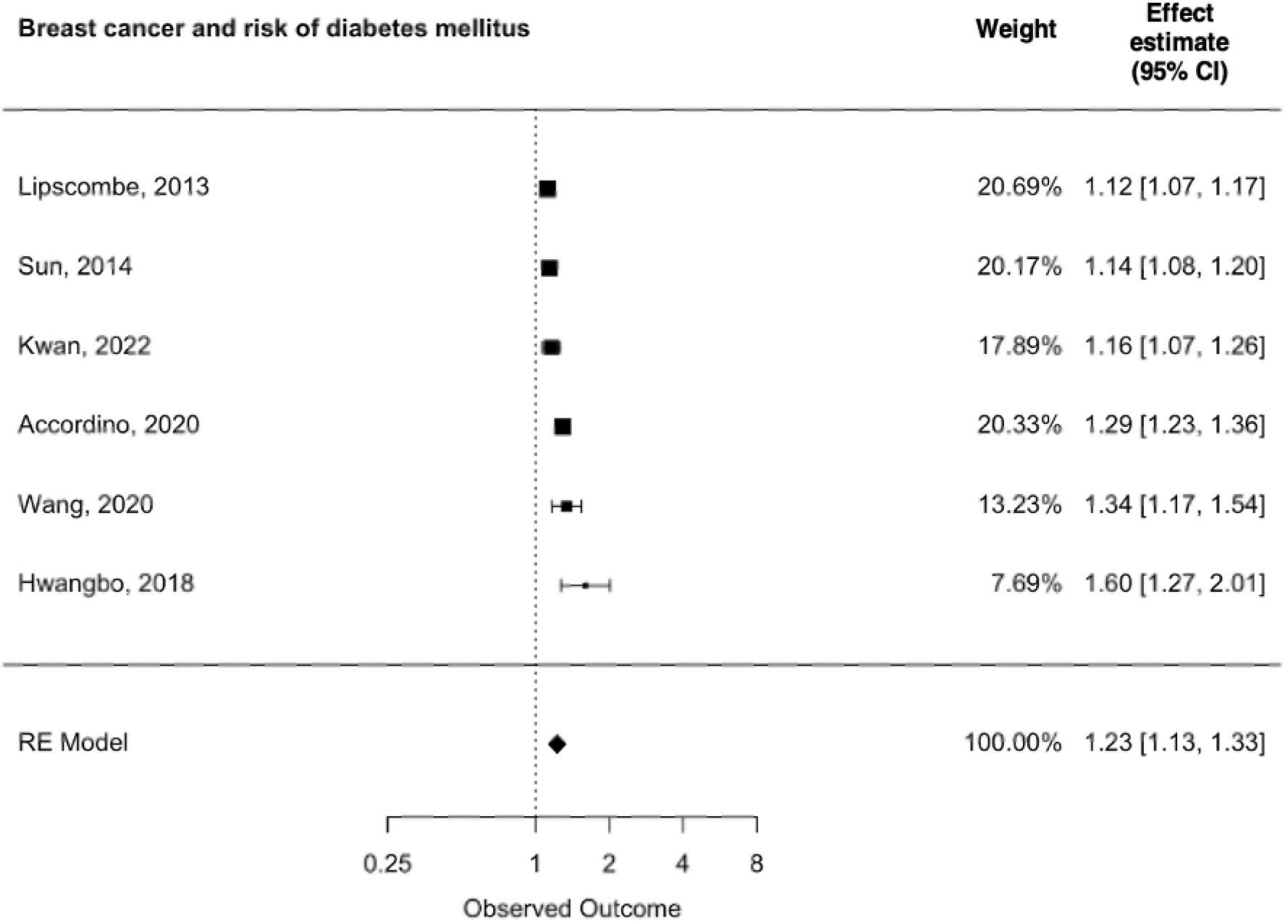
Breast cancer and risk of type 2 diabetes mellitus

### Study characteristics and risk of bias

Among the 15 eligible studies, 10 reported on T2D risk after either breast cancer, chemotherapy, or endocrine therapy [4, 6, 9, 21–23, 25–28]; five studies investigated more than one association [3, 5, 7, 20, 24]. The total number of breast cancer patients followed in cohort studies was 93,974; the size of the individual studies ranged from 114 to 24,976 patients [3–5, 7, 20–28]. The only case-control study included 1,445 breast cancer patients with T2D [6], while the only case-cohort study included 324 breast cancer patients with T2D [9]. Of the 15 studies, five were restricted to postmenopausal women—two studies based this on a study population of breast cancer patients aged over 55 years [3, 5], two restricted to breast cancer patients aged over 65 years [6, 28], and one restricted to breast cancer patients aged over 66 years [20]. Only four of the studies included information on weight in the adjusted analyses, either adjusting for overweight or body mass index, the latter as a continuous variable [4, 20, 23, 24]. For study characteristics, please see Table 1.

**Table 1:** Characteristics of the included studies

Study (reference no.)	Design and study period	Participants	Age (years)	Cancer characteristics	Follow-up	Association examined
Accordino, 2020, USA^(20)^	Cohort, 2005–2013	14,506 BC patients and comparison cohort of 13,529 persons matched by age and race	≥ 66	Stage I-III BC	≥ 2 years	Breast cancer vs. control^a^ Chemotherapy vs. control^b^
Choi, 2021, South Korea^(21)^	Cohort, 2008–2020	2522 BC patients with SERM use and comparison cohort of 2522 BC patients with no SERM use matched by propensity score	46.2 (mean)	Non-metastatic BC	7.3 years (mean)	Tamoxifen vs. control^b^
Hamood, 2018, Israel^(9)^	Case-cohort, 2002–2012	324 cases with DM and BC and a comparison subcohort of 448 BC patients (full cohort of 2246 BC patients)	55.5 (mean)	Early stage or regionally advanced. No in situ or metastatic BC	5.9 years (mean)	Endocrine therapy vs. control^b^ Tamoxifen vs. control^b^ Aromatase inhibitors vs. control^b^
Hwangbo, 2018, South Korea^(4)^	Cohort, 2003–2013	494,189 persons followed. 15,130 developed cancer, and 834 developed DM after cancer diagnosis. 1,434 BC patients included	–	–	7.0 years (median)	Breast cancer vs. control^a^
Ji, 2013, China^(22)^	Cohort, 2012	114 BC patients without DM. No comparison cohort	50.1 (mean)	–	3 to 72 months	Diabetes incidence among BC patients
Juanjuan, 2015, China^(23)^	Cohort, 2008–2014	1283 BC patients without DM and a comparison cohort of 1361 women without breast cancer	54.9 (mean)	–	41 months (mean)	Breast cancer vs. control^a^
Kwan, 2022, USA^(24)^	Cohort, 2005–2013	12,903 BC patients without DM and a comparison cohort of 64,862 women without breast cancer	61.2 (mean)	Invasive BC (all stages)	7.0 years (mean)	Breast cancer vs. control^a^ Chemotherapy vs. control^a^ Endocrine therapy vs. control^a^
Lipscombe, 2012, Canada^(6)^	Case-control, 1996–2008	1445 BC patients with DM and 7220 non-diabetic BC controls	≥ 65 (73.8 (mean))	Invasive BC (early stage)	5.2 years (mean)	Tamoxifen vs. control^b^ Aromatase inhibitors vs. control^b^
Lipscombe, 2013, Canada^(3)^	Cohort, 1996–2008	24,976 female BC patients and comparison cohort of 124,880 women matched by age	≥ 55 (68.5 (mean))	Invasive BC (early stage)	5.5 years (median)	Breast cancer vs. control^a^ Chemotherapy vs. control^a^
Ng, 2018, Australia^(25)*^	Cohort, 2003–2014	2421 female BC patients without DM and a comparison cohort of 24,210 women without BC and DM matched by age	Majority (87%) was ≥ 55	Prescriptions for endocrine therapy as proxy measurement for BC	4.9 years (median)	Endocrine therapy vs. control^a^ Tamoxifen vs. control^a^ Aromatase inhibitors vs. control^a^ Tamoxifen vs. aromatase inhibitor
Ng, 2018, Australia^(26)**^	Cohort, 2003–2014	3799 female BC patients without DM and a comparison cohort of 37,990 women without BC and DM matched by age	Majority (88%) was ≥ 55	Prescriptions for endocrine therapy as proxy measurement for BC	5.1 years (median)	Endocrine therapy vs. control^a^
Rao, 2020, India^(27)^	Cohort, 2017–2018	474 BC patients included. No comparison	Majority (80%) was ≥ 40	–	–	Diabetes incidence among BC patients
Santorelli, 2016, USA^(16)^	Cohort, 2007–2010	2678 BC patients and a comparison cohort of 8820 women matched by age	≥ 65	Stage I-III BC	2 years	Tamoxifen vs. control^a^ Aromatase inhibitors vs. control^a^
Sun, 2014, Taiwan^(7)^	Cohort, 2000–2011	22,257 BC patients and a comparison cohort of 89,028 persons	50 (mean)	–	7.73 years (median)	Breast cancer vs. control^a^ Tamoxifen vs. control^a,b^
Wang, 2020, Taiwan^(5)^	Cohort, 2001–2015	4607 BC patients and a comparison cohort of 23,035 women matched by age	≥ 55 (58.6 (mean))	–	Up to 15 years	Breast cancer vs. control^a^ Endocrine therapy vs. control^b^

^a^control with no breast cancer

^b^breast cancer control

^*^‘Incidence of comorbidities in women with breast cancer treated with tamoxifen or an aromatase inhibitor: an Australian population-based cohort study’

^**^‘Comorbidities in Australian women with hormone-dependent breast cancer: a population-based analysis’

### Breast cancer and subsequent risk of T2D

#### Study characteristics and risk of bias

Eight studies investigated the association between breast cancer and subsequent risk of T2D; all eight were cohort studies [3–5, 7, 20, 22–24]. Follow-up time ranged from three months [22] to a maximum of 15 years [5]. The study with three months follow-up included 114 patients and was excluded from the meta-analysis since it lacked a referent group. For the studies deemed eligible for inclusion in the meta-analysis, follow-up was therefore between two and 15 years. Referent groups in all studies were women without any previous cancer or T2D diagnosis, except for the study by Juanjuan where controls could potentially have another type of cancer [23]. Therefore, the study by Juanjuan was excluded from the meta-analytic models.

In all, 80,683 breast cancer patients were followed in the six studies included in the meta-analysis (Fig. 2). One study followed all types of cancer, but provided sparse information on their breast cancer cohort [4]. The remaining five studies acquired information on both cases and non-cancer controls from large registries with information on T2D diagnosis based on international classification of disease (ICD) diagnostic codes and/or from prescription claims for diabetes medications [3, 5, 7, 20, 24].

#### Meta-analyses

**Fig. 3:**
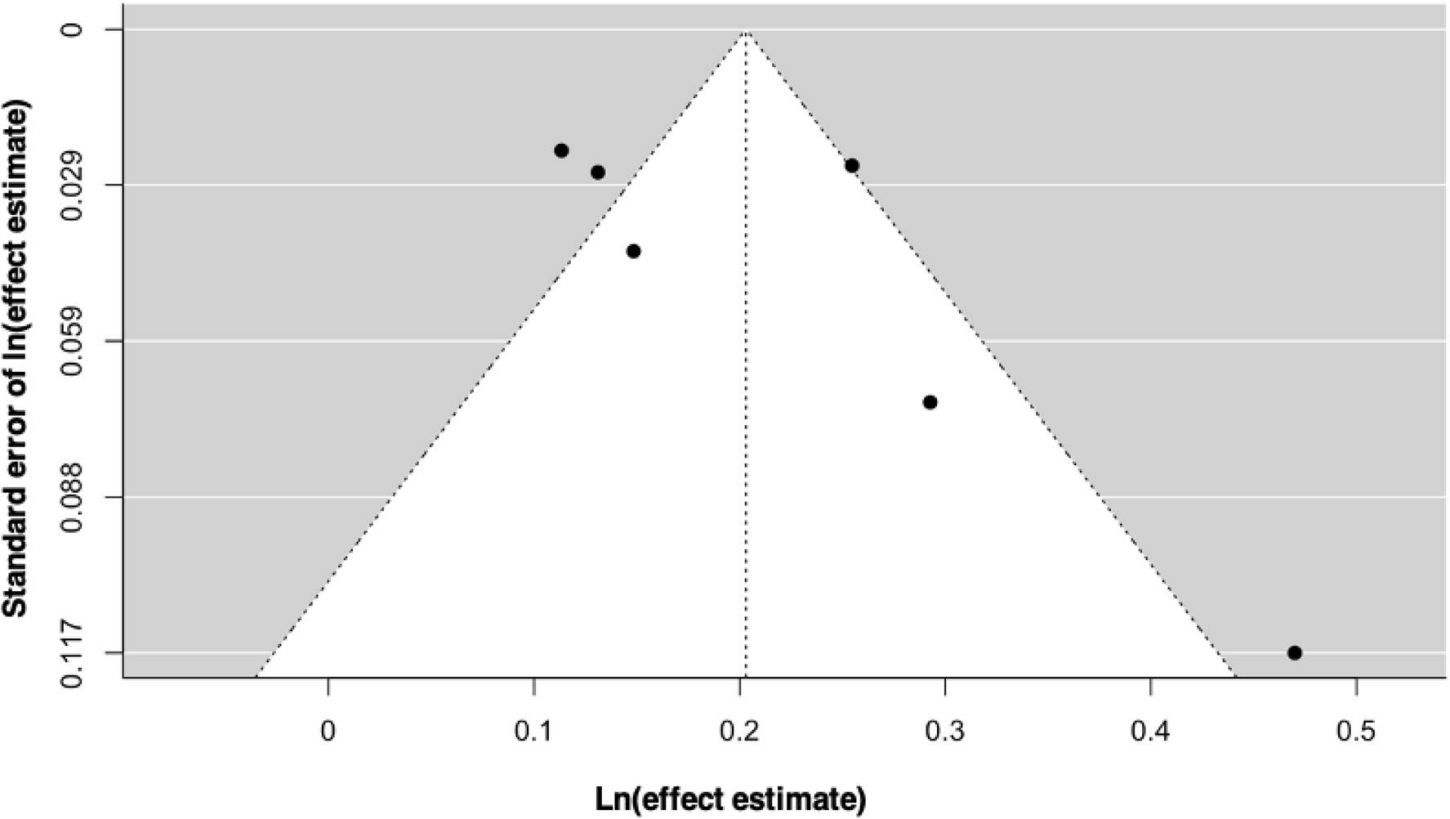
Funnel plot of studies pertaining breast cancer and risk of type 2 diabetes mellitus

The overall pooled EE for T2D among patients with breast cancer compared to non-cancer controls was 1.23 (95% CI: 1.13–1.33). There was evidence of statistical heterogeneity, as indicated by an overall I^2^ statistic of 97%. Nevertheless, because the studies consistently pointed in the same direction, we pooled the results using the REML. The small number of studies in the analysis weakened the validity of the funnel plot, but we did observe some evidence of publication bias (Fig. 3).

### Endocrine therapy and subsequent risk of T2D

#### Study characteristics and risk of bias

Nine studies reported on the association between endocrine therapy and subsequent T2D among breast cancer patients [5–7, 9, 21, 24–26, 28]. Of these, three reported on endocrine therapy in general without specifying tamoxifen or aromatase inhibitors [5, 24, 26], two reported on tamoxifen only [7, 21], two reported on tamoxifen and aromatase inhibitors separately [6, 28], and two reported on all three categories separately [9, 25]. Seven of these studies were cohort studies [5, 7, 21, 24–26, 28], one was a nested case-cohort study [9], and one was a case-control study [6].

Mean follow-up time in all but one study by Santorelli et al. [28], ranged from five to seven years. The study by Santorelli et al. had a maximum follow-up of two years which may not be a sufficient period of time for development of T2D attributable to endocrine therapy.

Except for one study, which included propensity score-matched controls [21], the studies standardized for age and baseline comorbidities, either by matching or adjusting. One study adjusted for smoking status [24].

#### Meta-analysis

**Fig. 4:**
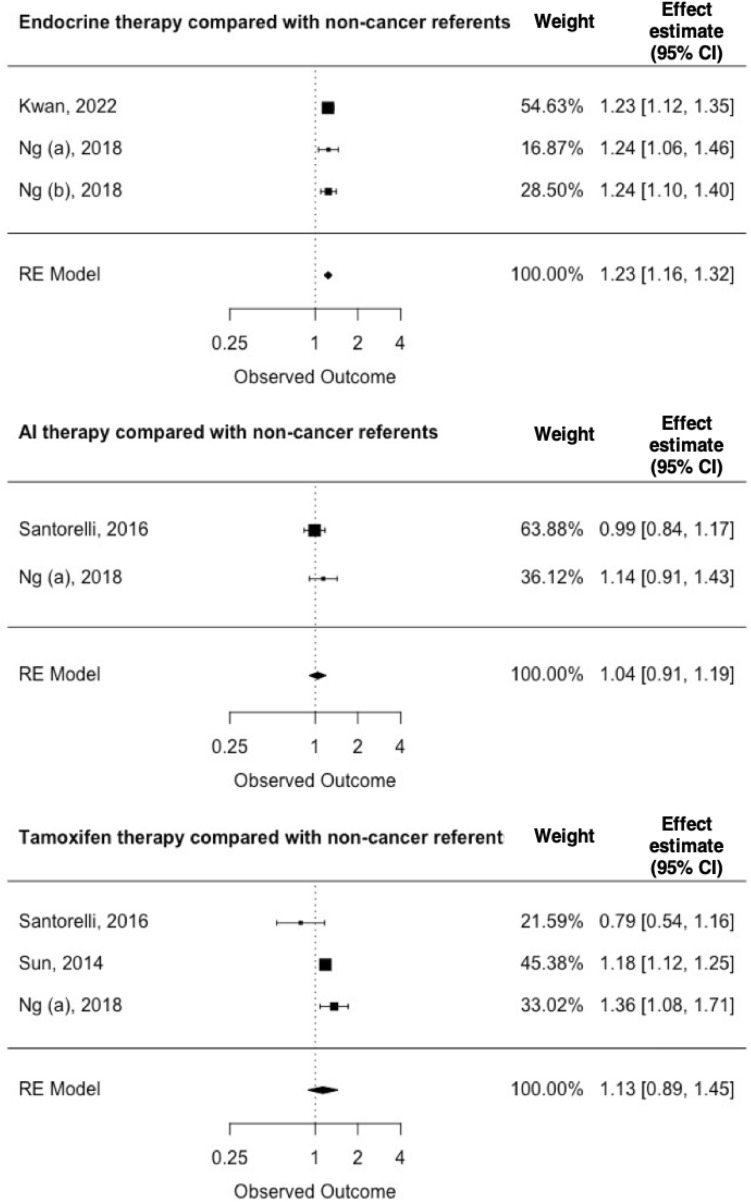
Breast cancer therapy and type 2 diabetes mellitus compared with non-cancer referents

**Fig. 5:**
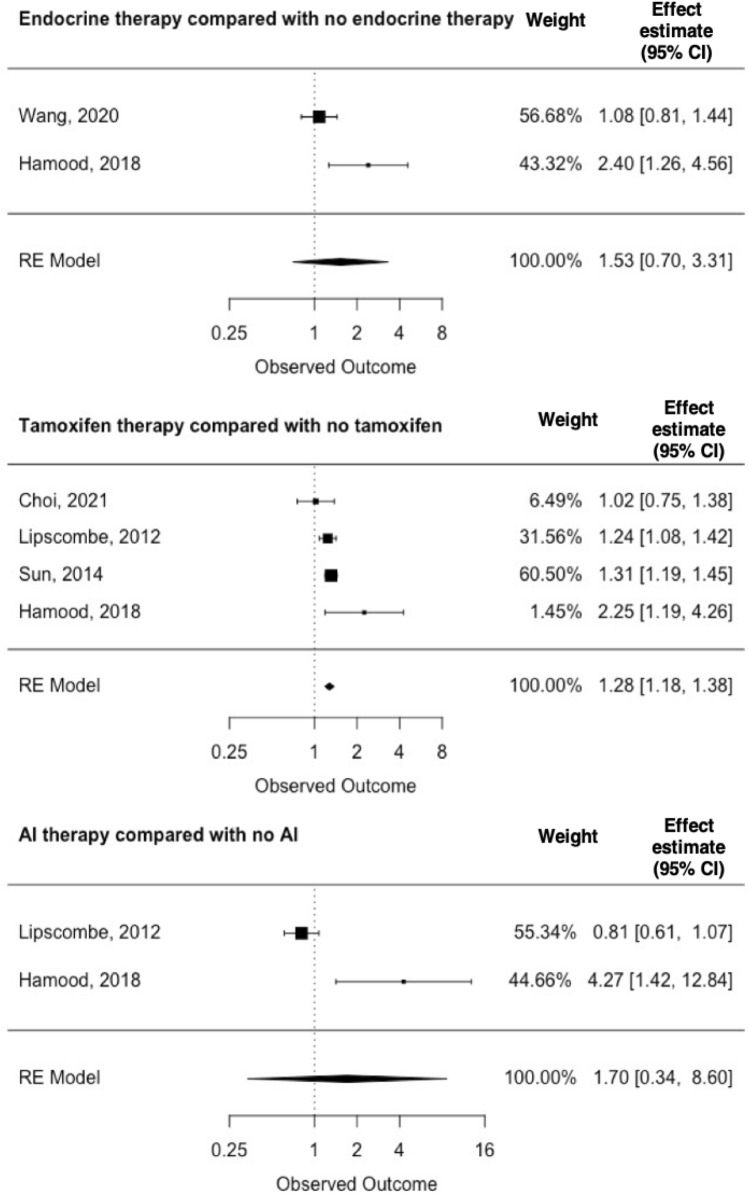
Breast cancer therapy and type 2 diabetes mellitus among breast cancer patients

Our findings on the incidence of T2D associated with the receipt of the various endocrine therapies are summarized in Figs. 4 and 5. One sub-analysis included four studies, two sub-analyses included three studies, and three sub-analyses included two studies. The low number of included studies impacts the precision of the pooled estimates. However, most of the included studies reported an increased risk of developing T2D, regardless of treatment and the type of comparison cohort (breast cancer patients who did not receive endocrine therapy, or non-cancer comparators). Breast cancer patients who received any endocrine therapy had an EE of 1.23 (95% CI = 1.16–1.32) compared with controls without cancer. In a sub-analysis of tamoxifen-treated breast cancer patients compared with breast cancer patients who had not received tamoxifen, pooling of the four included studies yielded an EE of 1.28 (95% CI: 1.18–1.38) with an I^2^ statistic of 0%.

### Breast cancer-directed chemotherapy and subsequent risk of T2D

#### Study characteristics and risk of bias

Four studies evaluated the association between chemotherapy and subsequent risk of T2D: all were cohort studies [3, 20, 24, 27]. Two included a comparison cohort of non-cancer controls [3, 24], one compared breast cancer patients treated with versus without chemotherapy [20]. One study did not include a reference group and instead investigated T2D incidence among the breast cancer patients examined at their clinic [27]. Still, none of the studies adjusted for concomitant glucocorticoid use which is prescribed with chemotherapy to counteract side effects, and can be associated with hyperglycemia [29]. Additionally, none of the studies adjusted for endocrine therapy. Because of the heterogeneity of the studies, we did not pool the results.

#### Results

Overall, the three studies with a comparison cohort showed an increased risk of T2D after chemotherapy, regardless of whether the controls were breast cancer patients or non-cancer referents. The study by Accordino et al. showed an adjusted OR of 1.19 (95% CI: 1.08–1.31) after at least two years follow-up [20]; in Lipscombe et al., the adjusted HR was 1.24 (95% CI: 1.12–1.38) after two years follow-up, which attenuated over 10-years of follow-up [3]. Kwan et al. had an adjusted HR of 1.23 (95% CI: 1.11–1.38) with a mean follow-up of seven years [24].

## Discussion

Our findings suggest that breast cancer survivors have increased risk of developing T2D compared with women without cancer. The elevated risk of T2D was particularly evident in the first few years after chemotherapy, dissipating in some studies with longer follow-up. Breast cancer patients who received tamoxifen therapy had higher risk of developing T2D compared with those who did not receive tamoxifen and compared with non-cancer referents.

Our study builds on the previous evidence presented in the meta-analysis by Ye et al. [16]. Yet, they restricted their meta-analysis to studies that examined the association between endocrine therapy and T2D and did not report associations for other breast cancer therapies. Furthermore, Ye and colleagues pooled data from one study on tamoxifen with data from studies investigating endocrine therapy in general, omitting other potentially eligible studies from their analysis [6, 28].

Our observed elevated risk of T2D associated with chemotherapy may be partly attributable to the afore-mentioned glucocorticoid-associated transient hyperglycemia [30]. We note that the studies investigating chemotherapy showed consistently increased risk of T2D, but the risk attenuated after treatment completion in at least one study [3]. This could be due to discontinuation of the chemotherapy-associated concomitant steroid use. However, none of the included studies incorporated information on glucocorticoid use, precluding the distinction of steroid-induced T2D from the effect of chemotherapy and chemotherapy-associated weight-gain per se [31]. Nonetheless, almost all breast cancer patients receive concomitant glucocorticoids during chemotherapy, so it is challenging to distinguish the effects of chemotherapy and glucocorticoids separately. Additionally, the results are potentially confounded by the receipt of endocrine therapy since none of the studies adjusted for this.

The increased risk of T2D in breast cancer survivors who received tamoxifen may be attributable to several factors. Tamoxifen has been associated with lower insulin sensitivity in premenopausal women with overweight, rendering them susceptible to developing T2D [15]. Mouse models suggest that tamoxifen exacerbates insulin deficiency by reversing estradiol-mediated protection of β-cells [14]. Accordingly, tamoxifen may increase the risk of T2D by reducing insulin sensitivity, and could also worsen the clinical course of T2D by promoting apoptosis in pancreatic β-cells. This may have clinical implications when selecting appropriate endocrine therapy for breast cancer patients with pre-existing T2D. Unfortunately, due to few studies, it was not possible to evaluate if the risk of T2D was higher after use of aromatase inhibitors as distinct from endocrine therapy in general.

Several issues warrant consideration when interpreting our findings. Most studies had small sample size, which impacted the sub-analyses investigating specific treatments. This is reflected in the wide confidence intervals in the forest plots illustrating the association of tamoxifen and aromatase inhibitor use with the risk of T2D. Another issue is the use of databases to obtain information on diabetes mellitus. Preclinical diabetes can remain undiagnosed for years; thus, the likelihood of testing and diagnosing T2D in a population of cancer survivors may be higher compared with their non-cancer counterparts. This would yield differential misclassification since breast cancer survivors may have more frequent health care contact and therefore be more likely to go to a hospital or primary care physician given their pre-existing condition. This may have inflated our observed risk of developing T2D in cancer survivors compared with their non-cancer counterparts. Another limitation is the lack of information on diabetes type which we assumed to be type 2 if not stated otherwise. We therefore set the age limit to 18 years, but some individuals aged above 18 years might still have been diagnosed with type 1 diabetes. Still, we expect this to be few women and to have negligible impact on our findings.

None of the included studies differentiated between breast cancer in pre- and postmenopausal women. This is important given the distinct disease courses and endocrine therapies recommended for pre- and postmenopausal women with breast cancer. Use of aromatase inhibitors became standard treatment for postmenopausal women in 2004 [32]. Yet, none of the studies overlapping this time period accounted for the updated clinical guidelines. Furthermore, no studies were restricted to premenopausal breast cancer patients. If the increased risk of T2D after breast cancer is a result of the anti-estrogen mechanisms of endocrine therapy, risk may be differential in premenopausal versus postmenopausal women due to the inherently higher estrogen-levels in premenopausal women. Such women receive tamoxifen therapy as guideline treatment for estrogen receptor-positive tumors and have the longest time at risk for subsequent development of T2D.

Although some of the studies adjusted for patient weight at baseline, no studies incorporated information on weight changes during follow-up. Breast cancer treatment leads to weight gain, which in turn, can also increase the risk of T2D [33]. Because of this, we cannot dismiss the possibility that our findings may be partly attributable to weight gain rather than a direct effect of breast cancer or its treatment on the risk of T2D.

Overall, our findings highlight the limited data on the association between breast cancer and subsequent risk of T2D. The statistical power varied across the studies and our meta-analyses. Moreover, we observed considerable heterogeneity across all our meta-analyses, precluding definitive conclusions.

Still, our findings provide insights into the potential impact of breast cancer and its treatments on the subsequent risk of T2D. Additional research is needed to fully understand these associations, particularly, the risk of T2D among premenopausal women with breast cancer, and the risk of T2D according to type of endocrine therapy—i.e., tamoxifen therapy or aromatase inhibitors. Our study highlights that more research is needed on the effects of radiation and anti-human epidermal growth factor receptor-2 (anti-HER2) therapies, which were not a focus in our study. We note that a single study suggested that left-sided radiation therapy was associated with increased risk of T2D, hypothesizing that the elevated risk was due to the proximity of the pancreas to the radiation field [24]. Anti-HER2 therapy is associated with heightened risk of cardiac toxicity, which is also associated with T2D [34]. Breast cancer patients at risk of both cardiovascular disease and T2D may have higher long-term morbidity, stressing the need for studies clarifying the impact of anti-HER2 therapy on the risk of T2D.

## Conclusion

In conclusion, this systematic review supports an association between breast cancer and an increased risk of T2D. Nonetheless, our review was limited by the low number of published studies and the potential for surveillance bias regarding T2D status in breast cancer survivors compared with their non-cancer counterparts. Furthermore, few studies reported data on the risk of T2D associated with specific breast cancer treatments, and no studies explicitly investigated the risk of T2D in premenopausal breast cancer patients. Still, the evidence points to increased risk of T2D in breast cancer survivors. Clinicians and those working in cancer survivorship care should consider the benefit of routine blood glucose testing in breast cancer survivors.

## Supplementary Information

Below is the link to the electronic supplementary material.

Online resource 1 (PDF 101.9 kb)

## Data Availability

Enquiries about data availability should be directed to the authors.
