# Supplementary material for: Breast cancer and incidence of type 2 diabetes mellitus: a systematic review and meta-analysis
Source: Breast Cancer Res Treat. 2023 Sep 1;202(1):11–22. doi: 10.1007/s10549-023-07043-6 (PMC10504120; doi:10.1007/s10549-023-07043-6)
Supplement: Supplementary file 1 — Online resource 1 (PDF 101.9 kb) [file 10549_2023_7043_MOESM1_ESM.pdf]

Breast Cancer and Incidence of Type 2 Diabetes Mellitus: A systematic review and meta-analysis

Journal: Breast Cancer Research and Treatment

Nanna Jordt[1], Kasper Kjærgaard[1], Reimar W. Thomsen[1], Signe Borgquist[2], Deirdre Cronin-Fenton[1]
[1]Department of Clinical Epidemiology, Department of Clinical Medicine, Aarhus University Hospital & Aarhus University, Denmark
[2]Department of Oncology, Aarhus University Hospital, Denmark

Corresponding author: Deirdre Cronin Fenton, Associate Professor, PhD, Department of Clinical Epidemiology, Department of Clinical Medicine, Aarhus University Hospital & Aarhus University, Denmark. E-mail: dc@clin.au.dk

Supplementary material

Online resource 1 Search terms

Search 1

Breast Neoplasms OR breast cancer OR mammary cancer AND Cancer Survivors OR surviv* AND Diabetes Mellitus OR hyperglycemic OR risk of diabetes OR diabetes risk OR diabetes incidence

Search 2

Diabetes Mellitus OR diabetes AND Breast Neoplasms OR breast cancer OR mammary cancer AND Tamoxifen OR hormone therapy OR Tamoxifen OR Drug Therapy OR chemotherapy OR aromatase inhibitor OR aromatase inhibitors OR Estrogen Antagonists OR Estrogen Antagonists
